# A Practical Treatise on Diseases in Children

**Published:** 1885-05

**Authors:** 


A Practical Treatise on Disease in Children. By Eustace Smith, Fellow of the Royal College of Physicians, Physician to His Majesty the King of the Belgians, Physician to the East London Children's Hospital, and to the Victoria Park Hospital for Disease of the Chest. New York: Wm. Wood & Co., 56 and 58 Lafayette Place. 1884.

In calling attention to this publication, we desire to extend to it something more than a mere expression of editorial courtesy. From a partial examination of its contents, we are prepared to give it the benefit of our endorsement, and to commend it to the favorable notice of the profession. Without attempting anything like an analysis of the work, we beg to refer to a few of its strong points; among which we would mention the author's views on hygiene, on the management of children, and on the administration of drugs, which we regard as wise and conservative.

The familiarity of the author with the leading characteristics of different diseases is shown in the portraitures he has drawn, giving the distinctive outlines of each by which they are recognized. This will prove one of the strongest recommendations of the work to public favor, as on no other head does the general practitioner meet so many perplexing difficulties as on the question of diagnosis, and from which the most familiar and best versed in the diseases of children are not exempt. In proof of the clearness with which he describes disease, we would refer to the articles on nervous and pulmonary troubles as examples of his striking delineations.

Without entering further into details, we simply add, that, in our opinion, the author has conferred a benefit upon the profession in presenting a complete treatise on this interesting branch of medical literature, and that it fills a very generally felt want, which will insure it a ready sale, and give it a front rank as a work of reference.

